# Spinocerebellar ataxia 27B: episodic symptoms and acetazolamide response in 34 patients

**DOI:** 10.1093/braincomms/fcad239

**Published:** 2023-09-10

**Authors:** Catherine Ashton, Elisabetta Indelicato, David Pellerin, Guillemette Clément, Matt C Danzi, Marie-Josée Dicaire, Céline Bonnet, Henry Houlden, Stephan Züchner, Matthis Synofzik, Phillipa J Lamont, Mathilde Renaud, Sylvia Boesch, Bernard Brais

**Affiliations:** Department of Neurology and Neurosurgery, Montreal Neurological Hospital and Institute, McGill University, Montreal, QC H3A 2B4, Canada; Department of Neurology, Royal Perth Hospital, Perth, WA 6007, Australia; Center for Rare Movement Disorders Innsbruck, Department of Neurology, Medical University of Innsbruck, Innsbruck 6020, Austria; Department of Neurology and Neurosurgery, Montreal Neurological Hospital and Institute, McGill University, Montreal, QC H3A 2B4, Canada; Department of Neuromuscular Diseases, UCL Queen Square Institute of Neurology and The National Hospital for Neurology and Neurosurgery, University College London, London WC1N 3BG, UK; INSERM-U1256 NGERE, Université de Lorraine, 54500 Vandoeuvre-les-Nancy, France; Service de Neurologie, Centre Hospitalier Régional Universitaire de Nancy, 54000 Nancy, France; Service de Génétique Clinique, Centre Hospitalier Régional Universitaire de Nancy, 54000 Nancy, France; Dr. John T. Macdonald Foundation Department of Human Genetics and John P. Hussman Institute for Human Genomics, University of Miami Miller School of Medicine, Miami, FL 33176, USA; Department of Neurology and Neurosurgery, Montreal Neurological Hospital and Institute, McGill University, Montreal, QC H3A 2B4, Canada; INSERM-U1256 NGERE, Université de Lorraine, 54500 Vandoeuvre-les-Nancy, France; Laboratoire de Génétique, Centre Hospitalier Régional Universitaire de Nancy, 54000 Nancy, France; Department of Neuromuscular Diseases, UCL Queen Square Institute of Neurology and The National Hospital for Neurology and Neurosurgery, University College London, London WC1N 3BG, UK; Dr. John T. Macdonald Foundation Department of Human Genetics and John P. Hussman Institute for Human Genomics, University of Miami Miller School of Medicine, Miami, FL 33176, USA; Division of Translational Genomics of Neurodegenerative Diseases, Hertie Institute for Clinical Brain Research and Center of Neurology, University of Tübingen, 72076 Tübingen, Germany; German Center for Neurodegenerative Diseases (DZNE), 72076 Tübingen, Germany; German Center for Neurodegenerative Diseases (DZNE), 72076 Tübingen, Germany; INSERM-U1256 NGERE, Université de Lorraine, 54500 Vandoeuvre-les-Nancy, France; Service de Neurologie, Centre Hospitalier Régional Universitaire de Nancy, 54000 Nancy, France; Service de Génétique Clinique, Centre Hospitalier Régional Universitaire de Nancy, 54000 Nancy, France; Center for Rare Movement Disorders Innsbruck, Department of Neurology, Medical University of Innsbruck, Innsbruck 6020, Austria; Department of Neurology and Neurosurgery, Montreal Neurological Hospital and Institute, McGill University, Montreal, QC H3A 2B4, Canada; Department of Human Genetics, McGill University, Montreal, QC H3A 0G4, Canada; Clinique des maladies neuromusculaires. Centre de Réadaptation Lucie-Bruneau, Montreal, QC H2H 2N8, Canada

## Abstract

Ashton C *et al* report a retrospective multi-centre cohort of 34 patients from Canada, France, Austria and Australia with spinocerebellar ataxia 27B, describing the common feature of episodic ataxia and other episodic features, as well as the inefficacy of acetazolamide in these patients.

## Introduction

We recently reported the phenotypic profile and natural history progression of 50 German patients^1^ with spinocerebellar ataxia 27B (SCA27B; MIM 620174), secondary to GAA repeat expansions in intron 1 of the fibroblast growth factor (*FGF14*) gene, confirming and extending previous reports showing that SCA27B is a late-onset, slowly progressive cerebellar syndrome.^2,3^ In addition, we detailed the treatment response to 4-aminopyridine (4-AP) in seven patients, including three prospective n-of-1 treatment experiences, suggesting symptomatic benefit of 4-AP in SCA27B.^1^ Both 4-AP and acetazolamide have been used therapeutically in episodic ataxia and shown to have a largely similar efficacy.^4–6^ Whether acetazolamide is also beneficial in SCA27B remains to be established. Here, we describe the real-world response to acetazolamide in cohorts of patients with SCA27B from Montreal (Canada), Innsbruck (Austria), Perth (Australia) and Nancy (France). We found acetazolamide to be only mildly beneficial in a minority of SCA27B patients and even precipitated episodic symptoms in one case. This observation of limited acetazolamide response may help inform management of patients with SCA27B.

## Materials and methods

A total of 107 patients with a pathogenic (GAA) _≥__250_ repeat expansion in the first intron of *FGF14* were identified across four sites, and the proportion of patients with recurrent episodic symptoms was recorded. The majority of the Montreal cohort (73/82), as well as the Perth and Nancy cohorts, have been previously reported. Phenotyping and treatment response were completed retrospectively from clinical records and, when possible, patient re-evaluation using a standardized data sheet.

Episodic symptoms were defined as the presence of a recognizable constellation of recurrent symptoms, which are intermittent, with clear onset and offset from the patient’s established baseline and can appear unprovoked or be induced, for example, by exercise or small amounts of alcohol. Patients must have episodic cerebellar symptoms (gait ataxia, dysarthria, diplopia, oscillopsia or appendicular ataxia) but could also have other episodic symptoms.

Treatment response was recorded in a non-controlled manner, defined as a subjective reduction in frequency or severity of symptoms, as judged by the non-blinded patient. The response was sustained when present on serial clinical reviews or non-sustained if there was waning of initial benefit on subsequent reviews.

The institutional review boards of the Montreal Neurological Hospital (MPE-CUSM-15-915), the Centre Hospitalier de l’Université de Montréal (ND02.045), the Medical University of Innsbruck (1022/2020), the University of Western Australia (RA/4/20/1008) and the Centre Hospitalier Régional Universitaire de Nancy (2020PI220) approved this study, and all patients provided written informed consent. The study complies with the Declaration of Helsinki.

The FGF14 repeat locus was genotyped as described previously.^2,7^ Repeat sizes were measured by capillary electrophoresis of fluorescent long-range PCR amplification products. The motif of the repeat expansion was analysed by targeted long-read nanopore sequencing in 59 cases (56 French–Canadian patients and 3 Australian patients), as described previously.^2^ Bidirectional repeat-primed PCRs targeting the 5′-end and the 3′-end of the locus were used to ascertain the presence of a GAA repeat expansion in the remaining cases. Expansions of at least 250 GAA repeat units were considered pathogenic.^2,3^ We identified one case in the Austrian cohort who was homozygous for (GAA)_264_ expansions. To exclude allele drop-out due to polymorphism at one of the primer-binding sites, we confirmed these initial findings using an alternate set of non-overlapping primers (forward: 5′-CCAGAGAGCAAATGACAGCA-3′; reverse: 5′-AGGCGCACTATATTGGGAAC-3′).

## Results

Episodic features were present in 86 of 107 (80%) patients across the four cohorts: Montreal 67/82 (84%), Innsbruck 9/10 (90%), Perth 2/3 (67%) and Nancy 8/12 (67%). Of these, 34 received acetazolamide in the clinical setting for treatment of episodic ataxia (Montreal *n* = 23, Innsbruck *n* = 7, Perth *n* = 1 and Nancy *n* = 3), with total daily doses ranging from 250 to 1250 mg. Clinical features of these 34 patients are shown in Table 1. At baseline, patients reported variable duration of episodes, from minutes to hours, with a frequency, when documented, of approximately daily (*n* = 9), weekly (*n* = 8) or quarterly (*n* = 2). Episodic symptoms included diplopia (23/34; 68%), vertigo (15/34; 44%), gait ataxia (26/34; 76%) and dysarthria (20/34; 59%). One patient experienced sudden falls or ‘drop attacks’, as well as episodic dysarthria. Chronic non-episodic cerebellar features included gait ataxia (33/34; 97%), appendicular ataxia (31/34; 91%), dysarthria (25/34; 74%), gaze-evoked nystagmus (20/34; 59%) and downbeat nystagmus (20/34; 59%).

**Table 1:** Demographics and clinical features of SCA27B patients treated with acetazolamide

	Montreal	Innsbruck	Perth	Nancy	Total
	*n* = 23	*n* = 7	*n* = 1	*n* = 3	*n* = 34
Female sex, no. (%)	9 (39%)	3 (43%)	0	2 (66%)	14 (41%)
Age at onset of episodic ataxia, years	52 (30–75)	56 (50–70)	48	62 (60–71)	56 (30–75)
Age at onset of permanent ataxia, years	60 (36–81)	58 (50–70)	48	65 (63–75)	59 (36–81)
Age at onset of walking aid, years	70 (46–88)	70 (55–76)	66	72 (65–78)	70 (46–88)
Age at last review, years	70 (46–88)	72 (59–82)	66	65 (65–78)	69 (46–88)
Repeat count of longer allele, repeat units	357 (285–508)^b^	377 (264–453)^b^	416	410 (334–492)^b^	364 (264–508)
SFDS score at last review	4 (3–6)	5 (2–6)	5	4 (3- 4)	4 (2–6)
**Episodic symptoms**					
Vertigo	10 (43%)	2 (29%)	0	3 (100%)	15 (44%)
Diplopia	16 (70%)	4 (57%)	0	3 (100%)	23 (68%)
Dysarthria	13 (57%)	6 (86%)	1	0	20 (59%)
Gait ataxia	16 (70%)	6 (86%)	1	3 (100%)	26 (76%)
Alcohol intolerance	11/21 (52%)	5/5 (100%)	1	2/2 (100%)	19/29 (66%)
Exercise induced	14/21 (67%)	6/6 (100%)	1	1 (33%)	22/31 (71%)
**Examination features**					
Gaze-evoked nystagmus	15 (65%)	3 (43%)	0	2 (66%)	20 (59%)
Downbeat nystagmus	17 (74%)	2 (29%)	0	1 (33%)	20 (59%)
Axial ataxia	22 (96%)	7 (100%)	1	3 (100%)	33 (97%)
Appendicular ataxia	20 (87%)	7 (100%)	1	3 (100%)	31 (91%)
Dysarthria	18 (78%)	5 (71%)	1	1 (33%)	25 (74%)
Pyramidal signs	3 (4%)	3 (43%)	1	0	8 (24%)
Ankle hyporeflexia	2 (9%)	1 (14%)	0	0	3 (9%)
Reduced pallaesthesia	6 (26%)	2 (29%)	0	2 (66%)	10 (29%)
**Investigations**					
Cerebellar atrophy on MRI	13/22 (59%)	5 (71%)	1	1/3 (33%)	20/33 (61%)
Neuropathy on NCS	2/8 (25%)	0/5	n/a	n/a	2/13 (15%)
SARA score,^a^ points	13.5 (1–34)	10 (8–25)	11	4.5 (4.5–7)	10.5 (1–25)
Disease duration at time of SARA score, years	17 (6–23)	10 (2–16)	18	4 (3–9)	13.5 (2–23)

Unless specified, data are reported as median (range).

a SARA scores available for 26 cases total: 15 Montreal, 7 Innsbruck, 1 Perth and 3 Nancy.

b Includes one patient with biallelic *FGF14* GAA repeat expansions: Montreal 304/292 repeats; Innsbruck 264/264 repeats; and Nancy 270/410 repeats.

NCS, nerve conduction studies; SARA, scale for the assessment and rating of ataxia; SDFS, spinocerebellar degeneration functional score.

### Treatment response to acetazolamide

**Figure 1:**
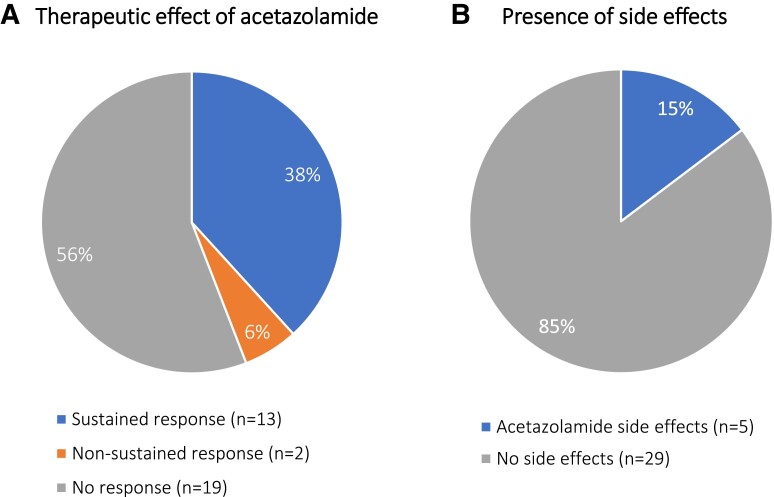
**Therapeutic effect and side effects of acetazolamide in patients with SCA27B.** (**A**) Proportion of patients (*n* = 34) responding to acetazolamide, where sustained response refers to improvement on serial clinic reviews and non-sustained response refers to waning of initial benefit on subsequent reviews. (**B**) Proportion of patients (*n* = 34) reporting side effects with acetazolamide.

A subjective improvement to acetazolamide was reported in 15 of 34 patients (44%), with 13 patients describing a sustained benefit (Fig. 1). No patients had complete cessation of episodic symptoms. Of the patients with sustained response, this was documented to be only a mild or partial improvement in six. Side effects were reported by five patients and, when specifically documented, included cognitive slowing (*n* = 1), subjective worsening of baseline ataxia (*n* = 1), dysgeusia (*n* = 1) and acral and/or peri-oral paraesthesia (*n* = 1), as well as one patient who had precipitation of severe episodes of ataxia. No serious adverse effects, including renal calculi, were reported.

Three patients (one French-Canadian and two Austrian) received 4-AP treatment, with two patients describing a subjective benefit, one of whom had a complete cessation of severe ataxic episodes.

One male French–Canadian patient had a trial of acetazolamide in the clinic. Prior to taking 250 mg acetazolamide orally, he walked 15 m in a time of 16.52 s. He then walked the same distance in 15.17 s, 45 min after taking acetazolamide, with self-reported improvement and clinician observed improvement in the appearance of his gait with fewer difficulties in turning. At the time of the in-clinic trial, he complained of mild peri-oral paraesthesia. The patient later experienced a severe episode of ataxia and dysarthria 2 h after taking acetazolamide. Severe attacks of ataxia were precipitated on three additional separate ingestions of the medication. Despite this, the patient elected to continue the medication on an as-needed basis and found that on days with subjectively worse ataxia, daily doses up to 1250 mg resulted in mild to moderate improvement of his symptoms without precipitation of severe episodes. However, the patient discontinued acetazolamide after 3 months due to intolerable acral paraesthesia.

### Innsbruck cohort

Of the total previously unreported 10 patients with SCA27B from Innsbruck, 7 (70%) were male and 5 (50%) had no family history of ataxia. Median *FGF14* GAA repeat expansion size was 377 repeat units (*range* 264–453 repeat units), with one patient homozygous for two expansions of 264 GAA repeats. All but one patient described recurrent episodic features (9/10; 90%), with an average age at onset of 57 years (*range* 50–70 years). Permanent cerebellar ataxia developed at 60 years (*range* 50–70 years) on average. Six patients reported caffeine triggering episodic ataxia (6/9; 67%). Other triggers included alcohol intake (6/9; 67%), exercise (6/9; 67%) and psychological stress (2/9; 22%). All patients had a pan-cerebellar syndrome with axial and appendicular ataxia, as well as dysarthria (8/10; 80%), gaze-evoked horizontal nystagmus (6/10; 60%) and downbeat nystagmus (4/10; 40%). Pyramidal signs were present in five patients (50%). An axonal length-dependent sensory neuropathy was confirmed by EMG in 2/7 (29%), with one also having motor involvement. Brain MRI showed cerebellar atrophy in eight patients (80%), which was limited to the vermis in five patients and extended to the hemispheres in three patients. The patient with homozygous (GAA)_264_ expansions had an earlier onset of disease and a more severe phenotype, requiring the use of a wheelchair at the age of 59, 9 years after disease onset, with a SARA score of 25. This individual had a partial response to acetazolamide 500 mg daily but had complete cessation of episodic symptoms after the addition of 4-AP 20 mg daily.

## Discussion

This cohort of patients was phenotypically similar to the cohort described by Wilke *et al.*,^1^ with a predominantly late-onset pan-cerebellar syndrome with frequent downbeat nystagmus (20/34; 59%), although with a higher frequency of episodic symptoms, which prompted acetazolamide treatment in 34 patients. Our findings suggest that SCA27B can frequently present with episodic symptoms, with 86 of 107 patients (80%) of our multi-centre cohort having documented episodic features. The relatively high frequency of episodic vertigo and diplopia, with or without downbeat nystagmus, may provide a diagnostic clue to SCA27B when assessing patients with late-onset ataxia. In addition to the previously described triggers of alcohol intake and physical exertion,^2,7^ we have documented that caffeine may precipitate attacks of ataxia in six Austrian patients, a finding that deserves further exploration.

In episodic ataxia type 2, acetazolamide has been shown in a randomized placebo-controlled trial to reduce episodes of ataxia by 48%, although it was associated with side effects in 45% of patients.^4^ A small retrospective cohort found that 29% of patients with episodic ataxia type 2 were non-responsive to acetazolamide.^6^ The episodic features of SCA27B do not seem to be as responsive to acetazolamide therapy, with 56% of patients in our cohort having no response. In comparison to the potential benefit of 4-AP in patients with SCA27B, as described by Wilke *et al.*,^1^ acetazolamide also appears to be less efficacious in those patients reporting improvement with this drug.

Our findings have the limitations of being retrospective and non-controlled, and the dosage of acetazolamide was not standardized, which may also explain our lower rates of side effects. Furthermore, treatment response was not measured with systematic or validated outcome measures across all four sites. Regardless, our real-world in-clinic experience on a large number of cases in different centres may inform clinicians of the likely failure of an empiric therapeutic trial of acetazolamide in patients with SCA27B. Clinical testing for SCA27B should further be considered for patients with late-onset episodic ataxia who do not benefit from acetazolamide. Our results, together with that of Wilke *et al.*,^1^ suggest that 4-AP might be the preferred symptomatic treatment in SCA27B.

## Data Availability

Data underlying this study are available upon reasonable request.
